# Reduced peripheral and respiratory muscle strength in pediatric patients after kidney transplantation

**DOI:** 10.1590/2175-8239-JBN-2022-0096en

**Published:** 2023-04-14

**Authors:** Michelle Hagi Frantzeski, Carolina Pacheco de Freitas Thomazi, Alexandre Severo do Pinho, Clotilde Druck Garcia, Janice Luisa Lukrafka

**Affiliations:** 1 Universidade Federal de Ciências da Saúde de Porto Alegre, Programa de Pós-Graduação em Ciências da Reabilitação, Porto Alegre, RS, Brazil.; 2 Centro Universitário Cenecista de Osório, Osório, RS, Brazil.; 3 Universidade Federal de Ciências da Saúde de Porto Alegre, Porto Alegre, RS, Brazil.; 4 Complexo Hospitalar Santa Casa de Porto Alegre-Hospital da Criança Santo Antônio, Departamento de Nefrologia Pediátrica, Porto Alegre, RS, Brazil.; 5 Universidade Federal de Ciências da Saúde de Porto Alegre, Departamento de Fisioterapia, Porto Alegre, RS, Brazil.

**Keywords:** Muscle Strength, Exercise Test, Transplantation, Pediatrics, Força Muscular, Teste de Esforço, Transplante, Pediatria

## Abstract

**Introduction:**

Reduced muscle strength and low-exercise capacity are well documented in adults, but there are few studies examining those impairments in children and adolescents after kidney transplantation. The objective of this study was to evaluate peripheral and respiratory muscle strength and the association with submaximal exercise capacity in children and adolescents after kidney transplant.

**Methods:**

Forty-seven patients between six and 18 years of age clinically stable after transplantation were included. Peripheral muscle strength (isokinetic and hand-grip dynamometry), respiratory muscle strength (maximal inspiratory and expiratory pressure), and submaximal exercise capacity (six-minute walk test – 6MWT) were assessed.

**Results:**

Patients had a mean age of 13.1 ± 2.7 years and an average of 34 months had elapsed since the transplantation. Flexors of the knee showed a significant reduction in muscle strength (77.3% of predicted) and knee extensors had normal values (105.4% of predicted). Hand-grip strength and maximal respiratory pressures (inspiratory and expiratory) also were significantly lower than expected (*p* < 0.001). Although distance walked in the 6MWT was significantly lower than predicted (*p* < 0.001), no significant correlation was found with peripheral and respiratory muscle strength.

**Conclusion:**

Children and adolescents after kidney transplantation have reduced peripheral muscle strength of knee flexors, hand-grip, and maximal respiratory pressures. No associations were found between peripheral and respiratory muscle strength and submaximal exercise capacity.

## Resumo

**Introdução:**

Força muscular reduzida e baixa capacidade de exercício encontram-se bem documentadas em adultos mas há poucos estudos examinando essas alterações em crianças e adolescentes após transplante renal. O objetivo deste estudo foi avaliar a força muscular periférica e respiratória e a associação com a capacidade submáxima de exercício em crianças e adolescentes após o transplante renal.

**Métodos:**

Foram incluídos 47 pacientes entre 6 e 18 anos de idade clinicamente estáveis após o transplante. Avaliou-se a força muscular periférica (dinamometria isocinética e de preensão manual), a força muscular respiratória (pressão inspiratória e expiratória máximas) e a capacidade submáxima de exercício (teste de caminhada de seis minutos – TC6M).

**Resultados:**

Os pacientes apresentaram média de idade de 13,1 ± 2,7 anos e uma média de 34 meses desde o transplante. Os flexores de joelho mostraram uma redução significativa na força muscular (77,3% do previsto) e os extensores de joelho apresentaram valores normais (105,4% do previsto). A força de preensão manual e as pressões respiratórias máximas (inspiratória e expiratória) foram significativamente inferiores ao esperado (*p* < 0,001). Embora a distância percorrida no TC6M tenha sido significativamente menor do que o previsto (*p* < 0,001), não encontramos nenhuma correlação significativa com a força muscular periférica e respiratória.

**Conclusão:**

Crianças e adolescentes submetidos ao transplante renal apresentam força muscular periférica reduzida de flexores de joelho e de preensão manual, bem como das pressões respiratórias máximas. Não foram encontradas associações entre força muscular periférica e respiratória e a capacidade submáxima de exercício.

## Introduction

Kidney transplant is an important therapeutic option for children with late chronic kidney disease (CKD)^1,2^. Typically, preemptive kidney transplantation has a low risk and high success rate and is associated with increased survival, with up to a 4-fold survival benefit compared with dialysis patients^3,4,5^. Over the past years, advances in immunosuppressive medication, surgical experience, and in-hospital care before and after transplant have improved patient and graft survival, the potential for growth, neurodevelopment, and quality of life^6,7^. Preemptive transplantation, before starting dialysis, seems to be the best therapy for children with end-stage renal disease (ERSD), offering the possibility of restoring normal renal function and eliminating many clinical manifestations of kidney disease^8,9^. However, this procedure also presents several side effects, especially among children, in whom immunologic responses are more intense^10^. Transplanted children are at higher risk of developing cardiovascular diseases, usually related to hypertension and dyslipidemia, which are already present in the CKD stage and persist after the transplant^11,12^. According to Chavers et al.^12^ the incidence of cardiovascular events in patients with stage 5 CKD was 24.3, 24.5, 23.9, and 36.9 in children aged 0–4 years, 5–9 years, 10–14 years, and 15–19 years, respectively. This risk is increased when associated with reduced exercise capacity^11^ and inactivity, leading to impaired functional capacity of children and adolescents after renal transplantation^13,14^.

Many children aren’t diagnosed with CKD until kidney function is already reduced and at an advanced stage. Because of this, osteopenia and musculoskeletal disorders can occur, leading to significant loss in muscle mass and strength^15,16^. Hogan et al.^17^ demonstrated impaired muscle strength in children exposed to CKD over a prolonged time. Pediatric kidney transplant recipients also have significantly reduced muscle strength and physical activity^18^.

Recent studies found evidence of systemic alterations in patients with chronic kidney disease, both adults and children. However, there is little evidence on peripheral and respiratory muscle strength after transplantation. We hypothesized that children and adolescents have reduced muscle strength and respiratory muscle strength after transplantation, which is directly associated with reduced exercise capacity. Therefore, the purpose of this study was to investigate peripheral and respiratory muscle strength after kidney transplantation in children and adolescents and its association with submaximal exercise capacity.

## Methods

This cross-sectional study was conducted with patients who received a kidney transplant during their follow-up period in the pediatric nephrology clinic at a reference center for transplantation in Porto Alegre, RS, Brazil. Parents or guardians were duly informed about the protocols and aims of the study. Informed consent or assent were obtained from participants before participation. The ethical committee of the Federal University of Health Sciences of Porto Alegre (UFCSPA-1503/11) and Irmandade Santa Casa de Misericórdia de Porto Alegre (ISCMPA-3506/1) approved the research protocol.

### Participants

Forty-seven children and adolescents (24 boys and 23 girls) between six to 18 years of age were selected after kidney transplantation (more than 30 days). Participants with neurological disease, acute or chronic orthopedic disease, and cognitive limitation were excluded. All participants were assessed during a scheduled follow-up visit to the outpatient clinic with the pediatric nephrology team.

### Procedures

After anthropometric and clinical data were recorded, patients were submitted to isokinetic dynamometry, hand-grip dynamometry, maximal respiratory pressure tests, and the six-minute walk test (6MWT). The sequence of tests had a minimum break time of 20 minutes. Patients who could not take the tests on the same day for personal reasons were assessed at the next visit. The sequence of tests was kept the same for all patients.

### Isokinetic Dynamometry and Hand-Grip Digital Dynamometry

The muscle strength of the knee flexors (KF) and elbow flexors (EF) and knee extensors (KE), and elbow extensors (EE) were assessed through the measurement of the peak torque (PT) of the dominant limb using an isokinetic dynamometer (BIODEX System 4 Pro^TM^, USA). Three attempts were made, and the limb selected at least twice was classified as dominant. To assess muscle strength, patients were placed on the dynamometer chair with the axis visually leveled with the axis of the articulation under study, which was immobilized to avoid compensations. The angular speeds for assessing the upper limbs were 90° and 120°/sec, with five repetitions^19^. For the lower limbs, angular speeds were 60° and 120°/sec, with 10 repetitions^20^. A 30-s break was given between each angular speed, and participants had a moment to accustom to the five movements for each measurement. All patients received verbal and visual stimuli throughout the test. The values were only compared with the predicted equation for the peak knee extensors and flexors torque at 60°/sec^21^.

We also used a handgrip digital dynamometer (Saehan Corporation^TM^, Korea). The individual was placed in a sited position with the shoulder abducted and in neutral rotation, the elbow supported in a 90° flexion, and the forearm and fist in a neutral position. Three measurements (in kilograms) were repeated with the dominant hand 30-seconds apart. The highest score was compared to the results provided by McQuiddy et al.^22^, which were normative data for grip strength in healthy children and young adults aged 6 to 19. Means and standard deviations were compared according to age and sex.

### Maximal Respiratory Pressure

Maximal respiratory pressures were measured with a manuvacuometer (GlobalMed MVD 300^®^, Porto Alegre, Brazil), a quick and non-invasive method to assess the strength of respiratory muscles, determined by the maximal inspiratory pressure (MIP) and maximal expiratory pressure (MEP). The test was conducted with the patient sited comfortably and performing at least three acceptable measurements—without leakage and with a duration of at least two seconds. Tests were repeated until no further improvements were obtained, and at least five technically satisfactory attempts differed by <10%. The highest value was used and expressed in centimeters of water (cmH_2_O). To analyze the predicted value, we used the reference for the pediatric population reported by Wilson et al.^23^.

### Six-Minute Walk Test (6MWT)

To assess the submaximal exercise capacity, the 6MWT was performed according to the guidelines of the American Thoracic Society^24^. The test was conducted in a 30-m corridor, and patients were instructed to walk as fast as possible for six minutes. Respiratory rate (RR) (counted as chest-wall expansions per minute), level of dyspnea, and fatigue of the lower limbs using a modified Borg scale were checked at the beginning, at the end, and during rest (one minute after the test). Other variables, such as heart rate (HR) and peripheral oxygen saturation (SpO_2_) were checked with a fingertip oximeter (Nonin Onyx^TM^ 9500, New Medical Inc, USA). The distance covered in the 6MWT was obtained in meters and compared with an equation for the normality of healthy children^25^.

### Statistical Analyses

Results were expressed as mean and standard deviation (symmetrical distribution) or median and interquartile range (asymmetrical distribution). Categorical variables were described in numbers (percentage). Normal distribution was confirmed using the Shapiro-Wilk test. Paired Student’s t-test (symmetrical distribution) or the Mann-Whitney test (asymmetrical distribution) was used. The existence of associations was assessed with the Spearman correlation test. Statistical analyses were performed using SPSS, Version 18.0 (SPSS, Inc., Chicago, IL, USA). The level of statistical significance was 5% (*p* ≤ 0.05).

## Results

Of the 52 potentially eligible patients, two did not fulfill the inclusion criteria, and three dropped out after the initial evaluation. In all, 47 patients with an average age of 13.1 ± 2.7 years from different parts of the country (89.4%) were assessed. The median time since transplantation was 34 (10–68) months, and the more prevalent diagnoses were kidney malformation (61.7%), glomerular disease (12.7%), hereditary cystic disease (6.4%), hemolytic-uremic syndrome (HUS) (6.4%), cortical necrosis (2.1%), and unknown cause (4.3%). According to the World Health Organization BMI z-score, 2 patients (4.3%) were underweight, 8 (17.0%) were overweight, and one (2.1%) was obese; the majority of patients (36, 76.6%) were classified as having normal weight.

Demographic and clinical variables are listed in Table 1. The average GFR^26^ was 79.38 ± 19.33 mL/min/1.73m^2^, with the majority of values between 60–89 mL/min/1.73m^2^, classified as stage two CKD (Kidney Disease Outcomes Quality Initiative – KDOQI). No patient was classified as grade 4 or 5 CKD. Patients were on optimal pharmacological therapy with an immunosuppression regimen; 97.9% used tacrolimus, 93.6% mycophenolate mofetil, and 70.2% prednisone. One-third used anti-hypertensive medication and 66% used other drugs.

**Table 1:** Clinical and anthropometric characteristics of patients undergoing renal transplant

Participant characteristics	n = 47
Gender (n)	
M/F	24/23
**Age (years)** *	13.1 ± 2.7 (7–18)
**Weight (kg)** *	45.2 ± 14.3 (24–77.8)
**Height (m)** *	1.47 ± 0.1 (1.13–1.75)
**BMI (kg/m^2^)** *	20.6 ± 4.3 (14–34.4)
Systolic blood pressure (mmHg)*	105 ± 10 (80–130)
**Diastolic blood pressure (mmHg)** *	63 ± 9 (40–80)
**Time post-transplant, n (%)**	
<6 m	9 (19.2%)
>6 m	38 (80.8%)
**Type of transplant, n (%)**	
Living related donor	23 (48.9%)
Deceased donor	24 (51.1%)
**Renal replacement therapy pre-transplant**	
Hemodialysis, n (%)	2 (4.2%)
Duration of hemodialysis (months)^‡^	96 (36–132)
Peritoneal dialysis n (%)	30 (63.8%)
Duration peritoneal dialysis (months)^‡^	6 (1–12)
HD and PD, n (%)	5 (10.7%)
None	10 (21.3%)
**GFR n (%)** ^†^	
≥ 90 (mL/min/1.73m^2^)	15 (32%)
60–89 (mL/min/1.73m^2^)	24 (51%)
30–59 (mL/min/1.73m^2^)	8 (17%)
**Laboratory values**	
Creatinine (mg/dl)*	1.1 ± 0.3 (0.6–2.0)
Sodium (mmol/L)*	140 ± 1.9 (134–143)
Potassium (mmol/L)*	4.4 ± 0.6 (1.2–5.3)
Hemoglobin (g/dL)*	12.6 ± 1.2 (10.7–15.9)
Creatine phosphokinase (U/L)^‡^	87 (62–144)
Urea (mg/dL)^‡^	42 (31–49)

* Values reported as mean ± standard deviation (minimum and maximum).

† Glomerular filtration rate calculated by the Schwartz formula^25^.

‡ Values reported as median and 25-75 percentiles. HD: Hemodialysis; PD: Peritoneal dialysis.

**Figura 1.**
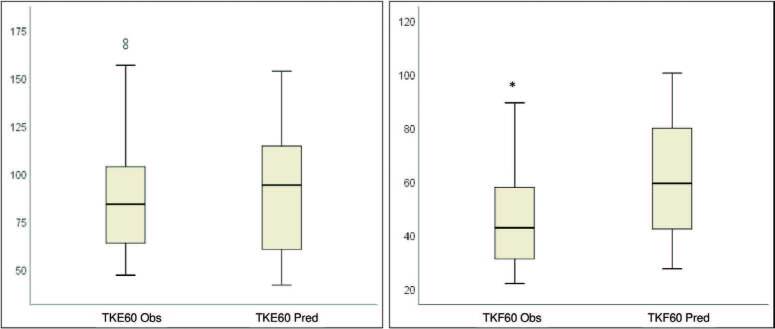
Values of peripheral muscle strength observed in isokinetic dynamometry in comparison with predicted values. *p = 0.000 compared with the achieved value. **TKE60 Obs:** observed value of torque knee extensors at 60°; **TKE60 Pred:** predicted value of torque knee extensors at 60°; **TKF60 Obs:** observed value of torque knee flexors at 60°; **TKF60 Pred:** predicted value of torque knee flexors at 60°.

Peripheral muscle strength variables are described in Table 2. Values of peak torque from flexors and extensors of the knee (angular speeds 60°) were compared with predicted values^21^. Knee flexors showed a significant reduction in muscle strength (77.3% predicted), and knee extensors had normal values (105.4% predicted) (Figure 1). The average hand-grip strength was significantly lower (*p* < 0.001) than predicted values from healthy subjects. In a subgroup analysis, boys had significantly higher strength scores in the upper limbs (EE90 *p* = 0.03, EF90 *p* = 0.006), handgrip (*p* = 0.03), and lower limbs (KE_60_ *p* = 0.05, KF_60_ *p* = 0.01, KE_120_ *p* = 0.005, KF_120_ *p* = 0.001) compared with girls.

**Table 2:** Isokinetic muscle force of upper and lower limbs and grip strength of transplanted patients

Isokinetic dynamometry (N-m/s) (n = 29)		
Torque peak elbow*		
Flexors	90º	21.7 ± 10.9 (15.3–24.8)
Extensors	90º	23.8 ± 8.8 (16.5–29.3)
Flexors	120º	17.4 ± 6.3 (13.3–21.7)
Extensors	120º	21.8 ± 8.8 (14.2–28.3)
Torque peak knee *		
Flexors	60º	45.4 ± 20.0 (30.0–58.1)
Extensors	60º	89.7 ± 36.7 (59.1–104.1)
Flexors	120º	40.6 ± 15.7 (27.3–52.0)
Extensors	120º	72.9 ± 29.2 (49.6–94.1)
Grip strength (kg)^†^		21.6 ± 7.9 (15.9–27.8)
Grip strength (kg)^†^ predicted		27.4 ± 8.6 (21.7–33.7)

* Values reported as mean ± standard deviation (minimum and maximum).

† 25 and 75 percentiles.

Maximal respiratory pressures (MIP and MEP) were also significantly lower than expected (*p* < 0.001). In submaximal exercise capacity, the distance covered in the 6MWT was significantly lower than predicted (*p* < 0.001) (Table 3).

**Table 3:** Obtained and predicted values of distance covered in the six-minute walk test (6MWD) and respiratory muscle strength

Variables		*p*
6MWD *	n = 47	
Distance covered (m)	499.9 ± 60.2 (388.0–661.0)	–
Distance predicted (m)	653.6 ± 63.2 (395.0–717.0)	<0.001^#^
**Maximal Respiratory Pressure** *	n = 44	
MIP (cmH_2_O)*	–55.2 ± 17.5 (–22.0 – –101.0)	–
MIP predicted (cmH_2_O)	–73.4 ± 12.8 (–54.0 – –111.1)	<0.001^#^
MEP (cmH_2_O)	62.8 ± 19.3 (18.0–112.0)	–
MEP predicted (cmH_2_O)	97.7 ± 16.9 (62.0–123.0)	<0.001^#^

* Values reported as mean ± standard deviation (minimum and maximum).

# Statistical significance between achieved and predicted values. MIP: maximal inspiratory pressure; MEP: maximal expiratory pressure.

Peripheral and respiratory muscle strength showed no significant correlation with exercise capacity (distance walked in the 6MWT) (Table 4). In a subgroups secondary analysis with patients submitted to renal replacement therapy before transplantation, there was no significant difference in exercise capacity and peripheral muscle strength between patients who did only peritoneal dialysis/hemodialysis and those who underwent preemptive transplantation. In addition, no correlation was found between peripheral and respiratory muscle strength and BMI z-score. However, positive and significant correlations were found between BMI and hand-grip and peripheral muscle strength of elbow flexors (90° and 120°), elbow extensors (90° and 120°), knee flexors (90° and 120°), and knee extensors 60° (data not showed).

**Table 4:** Correlations between functional capacity (6MWT) and peripheral and respiratory muscle strength

Variables	6MWT (m)	
	Correlation coefficient	*p* value
Flexors – elbow 90º	0.19	0.32
Extensors – elbow 90º	0.23	0.24
Flexors – elbow 120º	0.33	0.07
Extensors – elbow 120º	0.16	0.40
Flexors – knee 60º	0.16	0.40
Extensors – knee 60º	0.17	0.37
Flexors knee – 120º	0.08	0.66
Extensors – knee 120º	0.20	0.30
Grip strength (kg)	0.32	0.08
MIP (cmH_2_O)	–0.05	0.40
MEP (cmH_2_O)	0.22	0.14

MIP: maximal inspiratory pressure; MEP: maximal expiratory pressure.

## Discussion

Our study demonstrated that children and adolescents who underwent kidney transplantation have reduced peripheral muscle strength of knee extensors and respiratory muscle strength. However, we did not find any significant association between peripheral and respiratory muscle strength and submaximal exercise capacity.

In this study, we used the gold standard instrument to assess peripheral muscle strength, the isokinetic dynamometer in the upper and lower limbs. Krasnoff et al.^18^ showed, for the first time, results from the isokinetic dynamometer in 25 children after kidney transplantation and 11 adolescents after liver transplantation. The average values for strength of the knee extensors in both groups were very similar and significantly lower (67%) than the expected value for the age. In a second study^27^, the same group of researchers compared the results of the 25 kidney transplanted patients with 15 young people on dialysis: the muscle strength of the transplanted group was significantly higher than that of the dialysis group; however, patients did not reach normal levels.

Alayali et al.^28^ also found significantly lower quadriceps muscle strength in children on peritoneal dialysis than in controls. Although our measurements were at different velocities, we also found reduced quadriceps muscle strength at an angular velocity of 60°/sec, but only in flexors (77.3% of predicted values). Surprisingly, values of muscle strength during knee extension reached the expected values.

The exact mechanism of muscle strength reduction in these patients is still not clear. Some evidence points to multiple factors, including excess of toxins in the organism during CKD treatment, malnutrition, use of medications, metabolic acidosis (which may cause degradation of muscle proteins),^16,29^ and a systemic inflammation state^30^.

Peripheral muscle strength was significantly higher in boys at peak torque of the upper and lower limbs when assessed by an isokinetic dynamometer. Other researchers have found that peak torque of knee extensors is 30% higher in boys than in girls^18^. This better muscle performance in males may be associated with their higher muscle mass, a common characteristic in adolescents, and their higher capacity to generate tension as their muscles have a larger cross-sectional area^27^.

The children and adolescents in our study presented a significant decrease in MIP and MEP compared with the predicted values. It is well documented that adults and children^31,32,33^ with CKD experience skeletal muscle wasting and reduced exercise capacity. These alterations may persist even after renal replacement^18,27^. Ferrari et al.^13^ found a significant reduction in respiratory muscle strength of children and adolescents after transplantation compared with children in general. Persistent myopathy, mainly related to prior uremia and treatment with corticosteroids after transplantation, may be connected with this reduction^10^. Furthermore, patients often have sedentary habits, limiting the recovery of muscle and respiratory functions after the transplantation^34^.

A study that assessed bone structure, body mass, and muscle strength in 55 children and adolescents after kidney transplantation found a strong correlation (*r* = 0.73; *p* < 0.001) between the muscle cross-sectional area and muscle strength measured with the handgrip dynamometer^35^. Therefore, we presume that the low strength values obtained during the handgrip dynamometry, as found in our study, might be due to decreased muscle mass in kidney disease patients.

Our patients showed reduced submaximal exercise capacity, which may indicate that the effects of CKD persist after organ replacement. Other complications may arise, mainly related to surgery or medication^31,36^. Our patients covered 79% of the predicted values for age and sex-matched healthy individuals in the 6MWT. Several studies demonstrated reduced exercise capacity even after the transplantation^37,38^. A similar study by Ferrari et al.^13^ in children after kidney transplantation found values for the 6MWT of around 65% of the predicted value. This reduction is often related to an excessive protection by parents due to the chronic disease which, combined with frequent weight gain after transplantation, leads to physical inactivity, increasing the risk of cardiovascular diseases and other complications^35,39^. Cardiorespiratory fitness is considered a marker of cardiovascular health. Thus, children and youth with poor cardiovascular fitness have a risk factor for long-term health outcomes^39,40^.

In our study, we did not find correlations between functional capacity (6MWT) and peripheral and respiratory muscle strength, which may be due to the small sample size. However, similar data for the pediatric population are scarce. One study evaluated the relationship between muscle strength and 6MWD in children on peritoneal dialysis and showed a positive correlation between muscle strength and the 6MWD, indicating the close association between muscle strength and physical functioning tests^28^. Positive correlations between peripheral muscle strength and nutritional status were found only when using BMI, but longitudinal studies are necessary to explore these results. Respiratory muscle strength did not correlate with BMI or BMI z-score.

Our study had some limitations, such as the absence of a control group with healthy individuals and a very heterogeneous population with various time points post-transplant. We also mentioned the lack of reference values for peripheral muscle strength in the pediatric population, as only one study showed the prediction equation for peak torque of knee extensors and flexors at 60°/sec^21^. The level of physical activity was not evaluated because questionnaires and objective measurements were unavailable for patients below 12 years of age. Furthermore, another limitation of this cross-sectional study in children is the influence of puberty, body composition, and muscle structure; however, this is a limitation of the overall method.

In conclusion, children and adolescents submitted to kidney transplant have decreased knee flexors strength, hand-grip strength, and respiratory muscle strength. We did not find associations between muscle strength and submaximal exercise capacity. Based on our results, we suggest that children and adolescents should be appropriately evaluated and encouraged to participate in a rehabilitation program after kidney transplantation to restore their functional condition.
